# Efficient Red/Near‐Infrared‐Emissive Carbon Nanodots with Multiphoton Excited Upconversion Fluorescence

**DOI:** 10.1002/advs.201900766

**Published:** 2019-07-15

**Authors:** Kai‐Kai Liu, Shi‐Yu Song, Lai‐Zhi Sui, Si‐Xuan Wu, Peng‐Tao Jing, Ruo‐Qiu Wang, Qing‐Yi Li, Guo‐Rong Wu, Zhen‐Zhong Zhang, Kai‐Jun Yuan, Chong‐Xin Shan

**Affiliations:** ^1^ Henan Key Laboratory of Diamond Optoelectronic Materials and Devices Key Laboratory of Material Physics Ministry of Education School of Physics and Engineering Zhengzhou University Zhengzhou 450052 P. R. China; ^2^ State Key Laboratory of Molecular Reaction Dynamics Dalian Institute of Chemical Physics Chinese Academy of Sciences 457 Zhongshan Road Dalian 116023 China; ^3^ State Key Laboratory of Luminescence and Applications Changchun Institute of Optics Fine Mechanics and Physics Chinese Academy of Sciences Changchun 130033 P. R. China; ^4^ Institute of Atomic and Molecular Physics Jilin University Changchun 130012 P. R. China

**Keywords:** carbon nanodots, cellular imaging, fluorescence, multiphoton excitation, red/near‐infrared

## Abstract

Red/near‐infrared (NIR) emissive carbon nanodots (CNDs) with photoluminescence (PL) quantum yield (QY) of 57% are prepared via an in situ solvent‐free carbonization strategy for the first time. 1‐Photon and 2‐photon cellular imaging is demonstrated by using the CNDs as red/NIR fluorescence agent due to the high PL QY and low biotoxicity. Further study shows that the red/NIR CNDs exhibit multiphoton excited (MPE) upconversion fluorescence under excitation of 800–2000 nm, which involves three NIR windows (NIR‐I, 650–950 nm; NIR‐II, 1100–1350; NIR‐III, 1600–1870 nm). 2‐Photon, 3‐photon, and 4‐photon excited fluorescence of the CNDs under excitation of different wavelengths is achieved. This study develops an in situ solvent‐free carbonization method for efficient red/NIR emissive CNDs with MPE upconversion fluorescence, which may push forward the application of the CNDs in bioimaging.

It is accepted that red/near‐infrared (NIR) emissive nanoparticles have remarkable significance in bioimaging owing to their less photodamage to biological tissues, large tissue penetration, and high spatial resolution of light with long wavelength. Carbon nanodots (CNDs) have attracted much attention in recent years for their fascinating optical properties, low‐cost, biocompatibility, and so on,^1^, ^2^, ^3^, ^4^, ^5^ which have found great applications in bioimaging,^6^, ^7^, ^8^ sensing,^9^, ^10^, ^11^, ^12^ optoelectronics,^13^, ^14^, ^15^, ^16^ because of the above characters. Up to now, CNDs in blue and green spectral regions with photoluminescence (PL) quantum yield (QY) over 90% and 70% have been realized.^17^, ^18^, ^19^ Great efforts have been focused on red/NIR emissive CNDs,^20^, ^21^, ^22^, ^23^, ^24^, ^25^, ^26^, ^27^, ^28^ but the progress is still very limited. NIR windows (NIR‐I, 650–950 nm; NIR‐II, 1100–1350 nm; NIR‐III, 1600–1870 nm; NIR‐IV, centered at 2200 nm) excited fluorescence at red/NIR region and have great applications in deep tissue bioimaging.^29^ Nevertheless, the research on NIR windows excited red/NIR emissive CNDs is still rare. For example, Yang and co‐authors prepared NIR CNDs with two emission peaks of 665 and 710 nm and PL QY of 26%, 2‐photon excited fluorescence under 800 nm (NIR‐I) excitation has been demonstrated.^30^ Zhang and co‐workers prepared S and Se codoped CNDs and the CNDs have two emission peaks at 731 and 820 nm, 2‐photon excited fluorescence was also achieved under the excitation of 880 nm (NIR‐I), while the PL QY is only 0.2%.^31^ After that, Qu and co‐workers reported NIR emissive CNDs with emission peak at 760 nm and PL QY of 10%, and 1200–1400 nm (NIR‐II) excited 2‐photon and 3‐photon fluorescence were also demonstrated.^32^ However, the reported red and NIR emissive CNDs with multiphoton excited (MPE) fluorescence suffer from their low PL QY. In addition, NIR‐III window is the best excitation window for deep tissue imaging among all the NIR windows, but MPE fluorescence under NIR‐III excitation is still not yet achieved for CNDs. Thus, developing a new method for high efficient red/NIR emissive CNDs with NIR windows excited fluorescence will have a significant application foreground in deep tissue bioimaging.

Herein, red/NIR emissive CNDs with PL QY of 57% were prepared through a simple in situ solvent‐free carbonization method, which are the highest red/NIR emissive CNDs ever reported. The production yield of the CNDs is about 60%, and the longest wavelength emission peak of the CNDs is located at around 840 nm, and the PL covers all red region and most of the NIR‐I region. Exploiting the superior optical characteristics of the CNDs, 1‐photon and 2‐photon cellular imaging using the CNDs has been achieved in the B16‐F10 cells. Interestingly, the as‐prepared CNDs exhibit MPE upconversion fluorescence, and 3‐photon excited red/NIR fluorescence of the CNDs under excitation of 1600 nm (NIR‐III) has been demonstrated for the first time. Furthermore, 4‐photon excited red/NIR fluorescence of the CNDs is also achieved, indicating the CNDs have MPE upconversion fluorescence property.

The red/NIR emissive CNDs were prepared through an in situ solvent‐free carbonization method, which increases synthesis efficiency and reduces pressure of autoclave compared with conventional solvothermal method. The in situ solvent‐free carbonization synthesis of CNDs with red/NIR emission was carried out by mechanically mixing of o‐phenylenediamine (OPDA) and aluminum chloride hexahydrate (AlCl_3_ . 6H_2_O). After grinding for 10 min, the mixture was in situ carbonized for 12 h in an autoclave at 200 °C. After purification with water, the CNDs show bright red/NIR emission under UV illumination. Multibeam green rays were produced when the 532 light through the diffractive optical element (DOE) and multibeam red rays were observed when they passed through the red/NIR CND solution. The microstructure and preparation process of the DOE are show in Figures S1 and S2 (Supporting Information), and the preparation process of the CNDs was illustrated schematically in **Scheme** 1. Precursor OPDA was gone through polymerization reaction with the assistance of AlCl_3_ . 6H_2_O, and then dehydrogenation of the intermediate occurred under high temperature to form red/NIR emissive CNDs, as shown in bottom of Scheme 1. Detailed formation mechanism of the red/NIR emissive CNDs is discussed in next part, and production yield of the CNDs is about 60% (Figure S3, Supporting Information). Using the in situ solvent‐free carbonization method, the red/NIR emissive CNDs were easily prepared in large scale (up to 1500 mL, 1 mg mL^−1^), which lays a foundation for their practical applications, as shown in Figure S4 (Supporting Information). The as‐prepared CNDs were dispersed in ethanol or dimethyl sulfoxide (DMSO) to obtain red/NIR emissive CNDs.

**Scheme 1:**
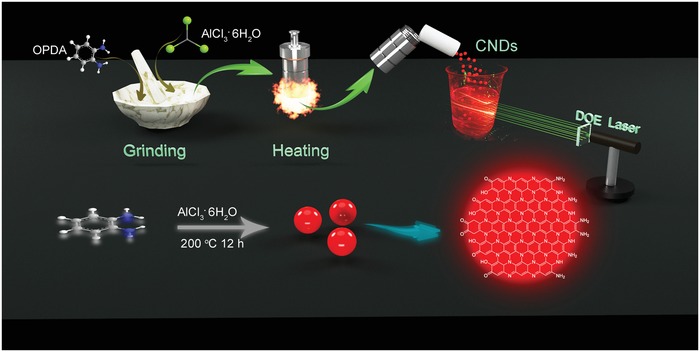
Schematics of the in situ solvent‐free carbonization process of the red/NIR emissive CNDs.

Transmission electron microscopy (TEM) and atomic force microscopy (AFM) are used to characterize the morphology and height of the CNDs. **Figure** 1a shows the TEM image of the CNDs, from which monodisperse CNDs can be observed. In the insets of Figure 1a, the statistics of particle size distribution (top left), high‐resolution TEM image (top right), and fluorescence image (bottom right) are shown. The size of the CNDs is in the range of 1.5–3.5 nm with an average size of about 2.5 nm. The lattice spacing of the CNDs can be determined to be about 0.21 nm from the high‐resolution TEM image, which corresponds to the d‐spacing of graphene (100) planes. Bright red/NIR emission can be observed from the fluorescence image of the CNDs, as indicated in the bottom right inset of Figure 1a. AFM image (Figure 1b) of the CNDs reveals that their height is in range of 3–4 nm (Figure 1c), which indicates the CNDs have a sphere‐like morphology. In order to investigate the inner structure of the CNDs, ^1^H nuclear magnetic resonance (^1^H NMR) and ^13^C NMR spectra were recorded, as shown in Figure 1d,e. In the ^1^H NMR spectrum, aromatic H centered at 8 ppm can be observed clearly. Other H signals from inner —NH (8.26–8.29 ppm), edge —NH_2_ (6.32–6.51 ppm), and —OH (10.21 ppm) are also detected. In the ^13^C NMR spectrum, the signals presented in the range of 110–140 ppm can be ascribed to sp^2^ conjugated aromatic C atoms.^25^ The signals from outside —C=O (196–197 ppm) are also detected. Figure 1f is the Fourier transform infrared (FTIR) spectrum of the CNDs, from which stretching vibrations of C—O (1132 cm^−1^), C—N (1301–1349 cm^−1^), C=C (1472 and 1501 cm^−1^), C=O/C=N (1577 and 1618 cm^−1^), and O—H/N—H (3200–3500 cm^−1^) can be observed. C—N stretching vibration at 1349 cm^−1^ indicates the form of N‐containing heterocyclic rings inside the CNDs. The vibration absorptions of the bonds suggest that the CNDs are mainly composed of polyaromatic structures with functional oxygen and nitrogen groups. Full X‐ray photoelectron spectroscopy (XPS) spectrum of the CNDs indicates that the primary elements are carbon, nitrogen, and oxygen, as shown in Figure 1g. High‐resolution spectrum of C1s shown in Figure 1h consists of three types of C: C—C/C=C (284.5 eV), C—N/C—O (285.5 eV), and C=N/C=O (287.1 eV). Two types of N including pyridinic N (399.3 eV) and amino N (400.7 eV) can be observed from the N1s spectrum shown in Figure 1i. In addition, two peaks located at 531.1 and 531.9 eV can be observed in the O 1s spectrum, as shown in Figure 1j, which can be assigned to the vibration of C=O and C—O/O—H.

**Figure 1:**
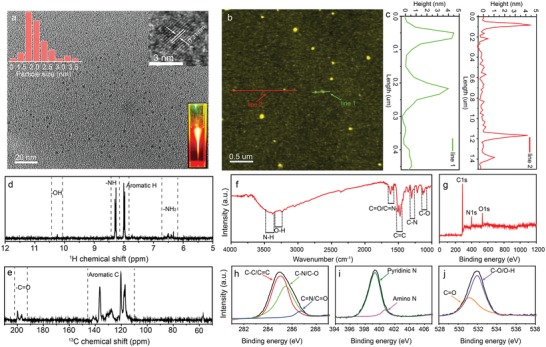
a) TEM image of the CNDs, the insets are the size distribution, HRTEM, and fluorescence images of the CNDs. b) AFM image of the CNDs. c) Height of the CNDs along the mark lines. d) ^1^H spectrum of the CNDs. e) ^13^C spectrum of the CNDs. f) FTIR spectrum of the CNDs. g) XPS spectrum of the CNDs. h–j) C1s, N1s, and O1s spectrum of the CNDs.

A possible formation mechanism for the high efficient red/NIR emissive CNDs is proposed based on the aforementioned analysis, as illustrated in **Scheme** 2. With the increase of temperature, aluminum chloride hexahydrate (AlCl_3_ . 6H_2_O) was first undergone hydrolysis to generate H_3_O^+^, HCl, and AlO_2_. A polymerization happened inside the OPDA with the assistance of AlCl_3_ and H_3_O^+^ under high temperature and high pressure. OPDA as single carbon source has been reported to generate green or yellow fluorescent CNDs,^20^, ^33^, ^34^ while no red emissive CNDs achieved due to small conjugated region. Then, the H_3_O^+^ can react to the polymeride through electrophilic reaction with the catalyst of AlCl_3_ under high temperature and pressure. At last, the intermediate polymeride was further in situ carbonized together with dehydrogenation reaction to form red/NIR emissive CNDs. Thus, the resulted CNDs possess efficient aromatic sp^2^‐conjugated system containing C=O, C—N, C=N, and O—H bands, which is favorable to red and NIR emission.^30^ The element contents of the CNDs are listed in Table S1 (Supporting Information), the ratio of element C and N is 2.57, which is close to the proposed chemical structure, indicating the proposed chemical structure is reasonable. For clear observation, the mixture precursor and resulted red/NIR emissive CND powders are also shown in the bottom of Scheme 2. To investigate the role of AlCl_3_ . 6H_2_O, different mole ratios between OPDA and AlCl_3_ . 6H_2_O of 5:1, 15:1, and 25:1 have been used to prepare CNDs, and the results are named CND‐1, CND‐2, and CND‐3, respectively. With the increase of AlCl_3_ . 6H_2_O, the prepared CNDs (1 mg mL^−1^) have enhanced absorption in long wavelength region (Figure S5, Supporting Information) due to enhanced sp^2^‐condugated system with the catalysis of AlCl_3_. The precursors OPDA and AlCl_3_ . 6H_2_O with mole ratio of 15:1 were treated 12 and 24 h, the enhanced absorption in long wavelength region can also be observed (Figure S6, Supporting Information).

**Scheme 2:**
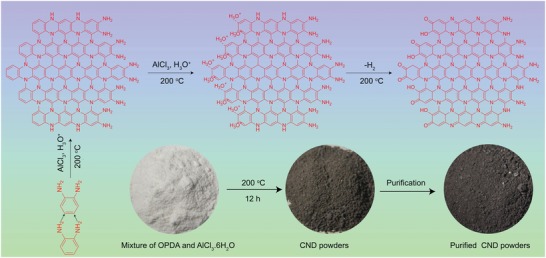
The formation process of the CNDs (top), followed by the mixture precursor and resulted CND powders (bottom).

The optical properties of the CNDs are investigated carefully via a home‐built PL measurement installation, as shown in **Figure** 2a. A 532 nm laser was used as the excitation source. The PL spectra of the CND‐1, CND‐2, and CND‐3 were measured, PL spectra of the CND‐2 with different concentrations are shown in Figure 2b and others are shown in Figure S7 (Supporting Information). The PL spectra show three obvious peaks at 590, 640, and 700 nm with a tail extending to 900 nm, which cover entire of red region and most of NIR‐I region. The insets in Figure 2b and Figure S4 (Supporting Information) are images of the CNDs with different concentrations under daylight condition. PL spectrum of CND‐1 has an enhanced intensity compared with CND‐2 and CND‐3 due to the enhanced sp^2^‐conjudagted system. While the PL intensity decreases in the region of 600–700 nm due to increase of absorption of CND‐1 in this region. The fluorescence of the CNDs can also be observed even under daylight excitation condition due to the high PL QY, as shown in Figure S8 (Supporting Information). The optimal condition for preparation of the red/NIR CNDs is that the mixtures with mole ratio of 15:1 are treated for 12 h under 200 °C. The absolute PL QYs of the CNDs under excitation of 490 nm are measured twice for accuracy, which are 56.98% and 54.75%, respectively (Figure S9, Supporting Information). CND‐2 with different concentrations was used as a sample to further study the optical properties of the CNDs in range of 750–900 nm. For clear observation, a band‐pass filter (BPF) was used to filter emission of CNDs in range of 600–700 nm, and the corresponding transmission spectrum of the BPF is shown in Figure S10 (Supporting Information). In the PL spectra (Figure 2c), three peaks of 760, 800, and 840 nm with a tail extending to 900 nm can be observed, which covers most of the NIR‐I window region. The first peak is caused by different transmissions of BPF, and the second and third peaks come from emission of the CNDs. Bright NIR emission can be observed after the filter of BPF, indicating that the CNDs have a strong emission with PL QY of 28.2% in NIR‐I region, as shown in the inset of Figure 2c. The polarization PL spectra of the CNDs are depicted in Figure 2d, and emission anisotropy of the CNDs can be observed (perpendicular represents 90° of excitation and emission), which is related to hot carriers recombination.^35^ The PL spectra at polarization angle from 0°–90° are shown in Figure S11 (Supporting Information). The plot of the CND polarization values is shown in the inset of Figure 2d, and the average polarization value is 0.11. Due to the large energy difference between excitation (2.33 eV) and emission (1.55 eV), the hot carriers experience a further relaxation process when they are excited by a linear polarization 532 nm laser. The relaxation process will randomize the momentum distribution of the carriers, leading to a small polarization value.

**Figure 2:**
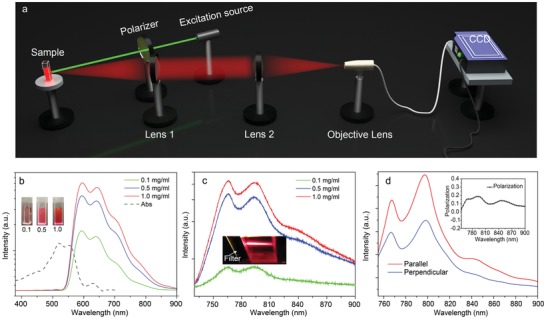
a) Schematics of the home‐built PL measurement installation for the CNDs. b) PL spectra of CND‐2 with concentration from 0.1 to 2.0 mg mL^−1^ under 532 nm laser illumination. c) Band‐pass filtered PL spectra of the CNDs with concentration from 0.1 to 2.0 mg mL^−1^ in the range of 750–900 nm under 532 nm illumination, the inset is the fluorescence image after the filter. d) Polarization PL spectra of the CNDs, the inset is the plot of polarization value.

To understand the PL mechanism including carriers' dynamics and energy relaxation dynamics of the CNDs; steady‐state spectra, time‐resolved spectra, and femtosecond transient absorption (TA) spectra have been recorded, as shown in **Figure** 3. Figure 3a is the contour plots of the CNDs as the excitation varies from 460–550 nm, and the CNDs exhibit excitation‐independent emission behavior. Few C—O—H/C—O—C groups of CNDs can account for this reason, which coincides with the preceding FTIR spectrum.^36^ The PL excitation (PLE) spectrum of the CNDs at different wavelengths was monitored, as shown in Figure 3b. PLE spectra at 590, 640, and 700 nm have identical peaks (marked with pool blue color), indicating the emission comes from the relaxation of the same excited state.^37^ In addition, the delay times of the CNDs at different wavelengths were recorded (Figure 3c), the area marked with gray is magnified in the inset. It is observed that the CNDs at 590, 640, and 700 nm have the same delay trend, the fitted lifetime is 3.42, 3.34, and 3.36 ns (Figure S12, Supporting Information), which indicates that the transition originates the same excited CNDs. TA spectra offer a useful tool to insight ultrafast dynamics of carrier relaxation and recombination.^38^, ^39^, ^40^ TA spectra with excitation at 540 nm, probe at 400–900 nm, with scan delay times from −0.5 to 1400 ps are shown in Figure 3d. Under 540 nm femtosecond pulse excitation, numerous ground‐state electrons are pumped into excited states leading to ground‐state bleaching (GSB) feature, and two GSB signals at 560 nm (GSB1) and 630 nm (GSB2) are observed, as shown in Figure 3e. There are three possible channels for electrons to depopulate: spontaneous emission, stimulated emission, and excitation state absorption.^41^, ^42^, ^43^ Compared with PL spectrum (Top of Figure 3e), the negative signal at about 700 nm generated by stimulated emission is detected in TA. TA kinetic traces of the CNDs probed at different wavelengths exhibit temporal evolution of GSB and stimulated emission process, as shown in Figure 3f. To investigate the relaxation process of the excited carriers, three exponent decay functions are used to fit the TA spectra. The fitted species‐associated difference spectra of the CNDs are shown in Figure S13 (Supporting Information). The times of the carriers of the CNDs are 1.49 ps, 148.03 ps, and 1.85 ns, respectively. A relaxation process model of carriers can be built by combining the polarization PL spectra, steady PL spectra, and TA spectra, as shown in Figure S14 (Supporting Information). After excitation, bits of hot carriers dissipate their energy in the same location while keeping the polarization; a large amount of hot carriers release their redundant energy by optical photon scattering within 1.49 ps. Then the relaxed electrons are trapped by surface states composed by carbon backbone and surface groups. 43% relaxed carriers in sp^2^ domain will undergo nonradiative transition to ground state (148.03 ps), and 57% relaxed carriers transition to ground state with the emission of red and NIR photons within 1.85 ns. The surface electron donating groups increase the electron cloud density of the CNDs in sp^2^ domain. The TA spectra provide a strong support that carbon cores and surface states play a key role toward high red and NIR emission of the CNDs.^44^

**Figure 3:**
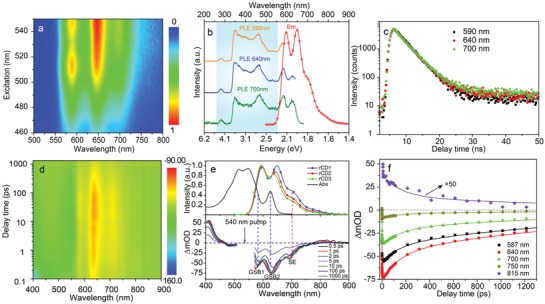
a) Contour plots of the CNDs as the excitation wavelength varies from 460–550 nm. b) PLE and PL spectra of the CNDs. c) Delay time of the CNDs at different wavelengths. d) 2D top‐view TA spectrum of the CNDs. e) Absorption spectrum, PL spectra (top) of the CNDs; and TA spectra (bottom) of the CNDs at indicated time from 0.5 to 1000 ps under 540 nm fs‐laser excitation. f) TA kinetic process of the CNDs probed at different wavelengths.

The superior optical property of the CNDs inspired us to investigate their application in cellular imaging. The cytotoxicities of the CNDs have been evaluated through the Cell Counting Kit‐8 (CCK8) assays before the cellular imaging experiment, as shown in **Figure** 4a. The cytotoxicity result shows that the murine melanoma cell (B16‐F10) viabilities can maintain over 90% after 24 h incubation with the CNDs, even the concentration of the CNDs is up to 100 µg mL^−1^. This confirms the good dispersibility, serum solubility, and low cytotoxicity of the CNDs. Then the cellular imaging experiment was conducted by using the CNDs as fluorescence agent. The bright‐field images of the B16‐F10 cells are shown in Figure 4b. For cellular imaging, the B16‐F10 cells were incubated with the CNDs for 24 h, the concentration of the CNDs is only 50 µg mL^−1^ due to the high PL QY. The fluorescence images of the cells are taken by one‐photon and two‐photon laser scanning confocal microscope. Figure 4c–e shows the fluorescence images of the B16‐F10 cells under excitation different wavlengths (552 and 638 nm in one‐photon excitation mode, 800 nm in two‐photon excitation mode). Bright deep red emission of the cells can be observed, indicating the CNDs are distributed into cytoplasm of the B16‐F10 cells uniformly. The corresponding merged images of fluorescence images and bight field images are shown in Figure 4f–h, and they coincide each other well. The result indicates that the CNDs are capable of red/NIR emissive fluorescence agent with low cytotoxicity.

**Figure 4:**
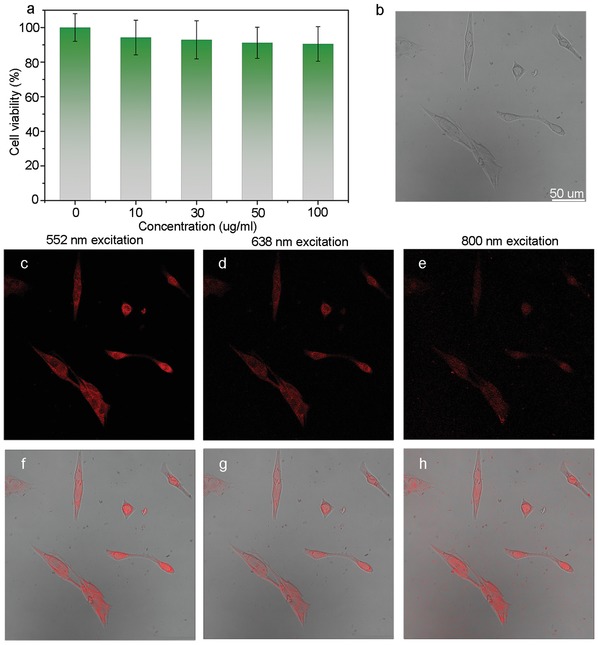
a) Cell viability of the B16‐F10 cells after incubation with CNDs of different concentrations. b) The bright‐field image of the B16‐F10 cells. c,d) The one‐photon fluorescence confocal images of the B16‐F10 cells under excitation of 552–638 nm. e) The two‐photon fluorescence confocal image of the B16‐F10 cells under excitation of 800 nm. f–h) The merged images of fluorescence confocal images and bright‐field images.

MPE upconversion fluorescence enables potential applications in deep tissue imaging; higher‐order MPE upconversion fluorescence have advantages of spatial confinement, increased penetration depth, reduced autofluorescence, and improved resolution over lower orders in bioimaging.^45^, ^46^, ^47^ There are several types of CNDs that have demonstrated MPE upconversion fluorescence, under 800–880 nm (NIR‐I) and 1200–1400 nm (NIR‐II) excitations.^26^, ^30^, ^32^, ^44^, ^48^ It has been reported that NIR‐III window is the best excitation window among the four NIR windows for its large penetration depth. Nevertheless, the emission of CNDs under excitation of NIR‐III window has not been reported before. To achieve effcient MPE upconversion fluorescence, D–π–A molecular architecture is one of the best choices for materials due to the large multiphoton absorption cross‐section.^49^, ^50^, ^51^ The as‐prepared CNDs have different kinds of functional groups within sp^2^‐conjugated aromatic system based on the above analysis, which may form a spectific donor–π–acceptor (D–π–A) archetecture (C—N, —NH_2_, and C—OH as electron‐donating groups; and C=O as electron‐accepting groups). To measure the MPE upconversionfluorescence of the CNDs, a setup of MPE upconversionfluorescence measurement system for the CNDs based on a femtosecond pulsed laser (fs‐laser) has been built, as illustrated in **Figure** 5a. The fs‐laser of 800 nm generated different wavelengths when the incident light passed through an optical parametric amplifier (OPA). Long‐pass filter was used to filter the light with short wavelength, and Glan prism was used to adjust the incident pulse energy. The lens were used to focus beam, and MPE upconversion fluorescence of the CNDs was coupled into a charge coupled device (CCD) by a objective lens. The fluorescence of the CNDs under 800 nm (NIR‐I) fs‐laser excitation has been obtained, as shown in Figure 5b. The fluorescence increases with the excitation power, and the slopes of the fluorescence intensity versus fs‐laser excitation fluence in ranges of 560–610 and 610–700 nm are 2.2 and 2.1, clearly indicating 2‐photon excited upconversion fluorescence (Figure 5c). Considering NIR‐III window is located in 1600–1870 nm, thus 1600 nm fs‐laser was used to investigate the MPE upconversion fluorescence properties of the CNDs. Figure 5d is fluorescence spectra of the CNDs under 1600 nm fs‐laser excitation, and the fluorescence intensity increases with the excitation fluence. A cubic dependence of integrated fluorescence intensity in ranges of 560–610 and 610–700 nm on excitation fluence can be observed, as shown in Figure 5e. To the best of our knowledge, this is the first time to achieve 3PE upconversion fluorescence of CNDs excited in a NIR‐III window. NIR‐III window is an optimal window for light penetration in deep tissues, which is also called Golden window. Interestingly, Figures 5f,g show the quartic dependence of the fluorescence intensity on excitation fluence under 2000 nm fs‐laser excitation for 4PE process. 4PE upconversion fluorescence of the CNDs was observed experimentally for the first time, which verify the favorable MPE upconversion fluorescence property. 1‐photon, 2‐photon, 3‐photon, and 4‐photon excited PL spectra of the CNDs are shown in Figure 5h, and no obvious shift can be observed. Figure 5i illustrates the 2‐photon, 3‐photon, and 4‐photon excitation process of the CNDs. The fluoresence images of the CNDs under different excitation wavelengths are listed in Figure 5j. Bright red/NIR fluorescecne can be observed, indicating the CNDs have favorable MPE fluorescence.

**Figure 5:**
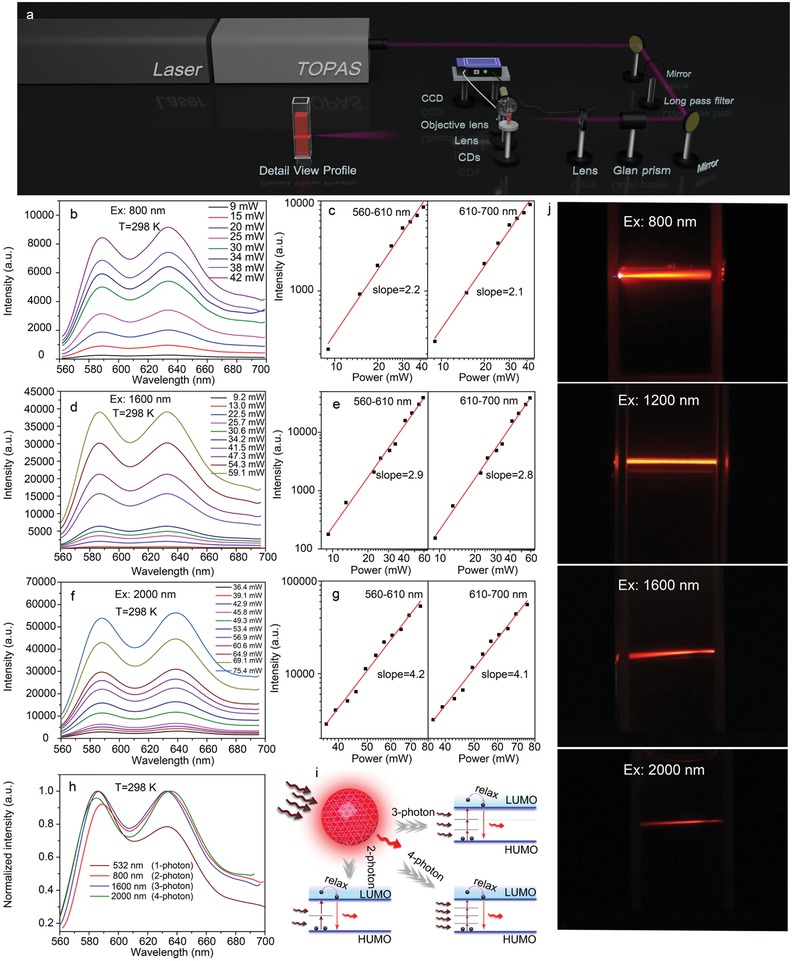
a) Setup of the MPE fluorescence mearsurement system for the CNDs based on a fs‐laser. b) PL spectra of the CNDs under 800 nm fs‐laser excitation with different excitation intensities. c) Square dependence of the integrated fluorescence intensity in ranges of 560–610 and 610–700 nm on excitation intensity. d) PL spectra of the CNDs under 1600 nm (NIR‐III) excitation with different excitation intensities. e) Cubic dependence of integrated fluorescence intensity in the ranges of 560–610 and 610–700 nm on excitation fluence. f) PL spectra of the CNDs under 2000 nm excitation with different excitation intensities. g) Quartic dependence of the integrated fluorescence intensity in the ranges of 560–610 and 610–700 nm on excitation intensity. h) 1‐,2‐,3‐, and 4‐PE PL spectra of the CNDs. i) MPE fluorescence process of the CNDs. j) Fluorescence images of the CNDs under 800–2000 nm fs‐laser excitation.

In conclusion, red/NIR emissive CNDs with PL QY up to 57% have been prepared through an in situ solvent‐free carbonization strategy for the first time. Detailed characterizations indicate that efficient sp^2^‐conjugated aromatic π systems are formed with the assistance of AlCl_3_ . 6H_2_O and H_3_O^+^, which is favorable for red/NIR emission. The CNDs show low cytotoxicity even at high concentration, 1‐photon and 2‐photon cellular imaging have been demonstrated by using the CNDs as fluorescence agent. MPE upconversion red/NIR fluorescence of the CNDs has been achieved, and the formed spectific D–π–A architecture within the CNDs is helpful for MPE upconversion fluorescence. In addition, 3‐photon excited red/NIR fluorescence of the CNDs under 1600 nm (NIR‐III) excitation has been demonstrated, and 4‐photon excited red/NIR fluorescence of CNDs is also observed experimentally for the first time. Benefitting from the efficient red/NIR emission and MPE upconversion fluorescence of the CNDs under excitation of NIR windows, this work demonstrates a simple method for developing highly efficient red/NIR emissive CNDs, which may push forward application of the CNDs in bioimaging.

## Experimental Section

*Chemicals*: The reagents in this work are o‐phenylenediamine (C_6_H_8_N_2_, purity >99.5%), aluminum chloride hexahydrate (AlCl_3_ . 6H_2_O, purity >99.5%), dimethylsulfoxide, absolute ethyl alcohol, which were used as received without purification. All of the chemicals were purchased from Shanghai Maclean Biochemical Technology Co., Limited. The water used to wash the sample was purified by an ultrapure water machine of Waterjet Laboratory.

*Preparation of Red/NIR Emissve CNDs*: The red/NIR emissive CNDs were prepared via an in situ solvent‐free carbonization method and all of the processes are as follows: 5 mmol o‐phenylenediamine and 0.33 mmol AlCl_3_ . 6H_2_O were ground uniformly in a mortar for 10 min. Then the mixture was transferred to a 30 mL autoclave for heating 12 h at 200 °C. After that, the autoclave was taken out and cooled to room temperature naturally. The obtained carbonized powders were washed with deionized water three times to remove impurities, and the remnants were dried in an oven with 60 °C. In the end, the dried powders were dissolved into ethanol or DMSO to achieve red/NIR emissive CNDs for further characterizations. For CND‐1 and CND‐3, the ratio between o‐phenylenediamine and AlCl_3_ . 6H_2_O are changed to 5:1 and 25:1.

*Cell Culture*: B16‐F10 melanoma cells were obtained from the Chinese Academy of Sciences Cell Bank (Shanghai, People's Republic of China). They were incubated in Roswell Park Memorial Institute (RMPI) 1640 medium containing 10% fatal bovine serum (FBS) and 1% antibiotics (penicillin–streptomycin, 10 000 U mL^−1^), respectively. Then the cells were cultured in 5% CO_2_ atmosphere at 37 °C.

*Cytotoxicity Measurements*: The cytotoxicity was measured via CCK8 assay. B16‐F10 cells were cultured on 96‐well plates. After 24 h, CNDs with different concentrations were added into each well. After further incubation for 24 h, 10 µL of the CCK8 solution was added to each well of the plate. The absorbance was measured using a microplate reader (BIO‐RAD 550) after 2 h. The cell viabilities (%) were calculated as follows

(1) $$\text{Cell}\;\text{viability}\;\left(\%\right)\;=\;{\text{OD}}_{\text{Treated}}{\text{/OD}}_{\text{Control}}$$

where OD_Control_ was obtained in the absence of CNDs, and OD_Treated_ was obtained in the presence of CNDs, respectively.

*Cellular Imaging*: B16‐F10 melanoma cells were incubated in RMPI 1640 medium containing 10% FBS and 1% antibiotics. Suspensions of CNDs (50 µg mL^−1^) from the stock solution were prepared with DMSO. The suspension was sonicated for 10 min to complete dispersion. 1 mL of the suspension was added to the medium, and then the cells were incubated at 37 °C in a 5% CO_2_ incubator for 12 h. Prior to fixation of the cells on the slide for inspection with a fluorescence microscope, the excess CNDs were removed by washing 3 times with warm phosphate buffer saline. The cellular imaging pictures were taken at a Leica TCS SP8 STED 3X laser scanning confocal microscope.

*Characterization*: The TEM and HRTEM images of the CNDs were pictured by a FEI Tecnai G2‐F20 TEM at 200 kV. AFM images were taken by a Multimode 8 instrument. XPS measurements were performed using s Thermo ESCALAB 250 spectrometer. The element analysis experiment was conducted by using a Vario EL III elemental analyzer. The radicals of the CNDs were characterized by a Bruker VERTEX‐70 FTIR spectrometer. The 2D PL spectra and UV–vis were recorded by a Hitachi F‐7000 and Shimadzu UV‐3101 PC spectrophotometer, respectively. The absolute PL QY was obtained by a calibrated integrating sphere in FLS920 spectrometer, and fluorescence lifetimes were characterized using FLS920 time‐corrected single photon counter system. The cellular imaging pictures were taken at a Leica TCS SP8 STED 3X laser scanning confocal microscope.

*Transient Absorption Spectroscopy*: A Ti:sapphire laser (Spectra‐Physics, Spitfire ACE, 800 nm, 4.5 mJ pulse−1, full width half maximum (fwhm) 35 fs, 1 kHz) was used to perform the ultrafast transient absorption measurements. In this experiment, pump pulses at 540 nm were generated through an automated OPA (Spectra‐Physics, TOPAS Prime). The white‐light probe was generated by focusing the fundamental laser output on a CaF2 window. The pump pulses were chopped to 25 Hz through a synchronized chopper (Newport, Model 3502). The probe pulse was focused into optical fiber that is coupled to a spectrometer (Ocean Optics, QE PRO) after passing through the sample. The near infrared CNDs with concentration of 1 mg mL^−1^ were placed in a 2 mm optical length quartz cuvette. The group velocity dispersion effect of solvent was corrected by a chirp program.

*Multiphoton Excited Fluorescence Spectroscopy*: A regenerative amplified femtosecond Ti:Sapphire laser system (Coherent, Legend, 800 nm, 7.1 mJ pulse^−1^, ≈30 fs, 1 kHz) was used to perform MPE fluorescence experiment. Pump pulses of 1600 and 2000 nm were generated through an automated optical parametric amplifier (Light Conversion, TOPAS Prime) pumped by the femtosecond laser system. The multiphoton fluorescence signal was acquired at the left side of an excited near‐infrared CNDs placed in 1 cm optical length quartz cuvette, and the signal is focused into a spectrometer (ANDOR, Shamrock 303i) coupled with CCD (ANDOR, Newton DU920P) through an optical fiber. A long‐pass filter was employed to filter out the scattered light at excitation pump laser frequencies.

## Conflict of Interest

The authors declare no conflict of interest.

## Supporting information

Supplementary
