# Supplementary material for: Efficient Red/Near‐Infrared‐Emissive Carbon Nanodots with Multiphoton Excited Upconversion Fluorescence
Source: Adv Sci (Weinh). 2019 Jul 15;6(17):1900766. doi: 10.1002/advs.201900766 (PMC6724478; doi:10.1002/advs.201900766)
Supplement: Supplementary file 1 — Supplementary [file ADVS-6-1900766-s001.pdf]

Copyright WILEY-VCH Verlag GmbH & Co. KGaA, 69469 Weinheim, Germany, 2019.

ADVANCED SCIENCE
Open Access

Supporting Information

for *Adv. Sci.,* DOI: 10.1002/advs.201900766

Efficient Red/Near-Infrared-Emissive Carbon Nanodots with Multiphoton Excited Upconversion Fluorescence

Kai-Kai Liu, Shi-Yu Song, Lai-Zhi Sui, Si-Xuan Wu, Peng-Tao Jing, Ruo-Qiu Wang, Qing-Yi Li, Guo-Rong Wu, Zhen-Zhong Zhang, Kai-Jun Yuan, and Chong-Xin Shan**

Supporting Information

Efficient Red/Near-Infrared-Emissive Carbon Nanodots with Multiphoton Excited Upconversion Fluorescence

Kai-Kai Liu, Shi-Yu Song, Lai-Zhi Sui, Si-Xuan Wu, Peng-Tao Jing, Ruo-Qiu Wang, Qing-Yi Li, Guo-Rong Wu, Zhen-Zhong Zhang*, Kai-Jun Yuan* and Chong-Xin Shan**

Dr. K. Liu, Mr. S. Song, Miss S. Wu, Prof. Z. Zhang, Prof. C. Shan
Henan Key Laboratory of Diamond Optoelectronic Materials and Devices, Key Laboratory of Material Physics, Ministry of Education, School of Physics and Engineering, Zhengzhou University, Zhengzhou, 450052, People's Republic of China.
E-mail: cxshan@zzu.edu.cn
Dr. L.-Z. Sui, Prof. G.-R. Wu, Prof. K.-J. Yuan
State Key Laboratory of Molecular Reaction Dynamics, Dalian Institute of Chemical Physics, Chinese Academy of Sciences, 457 Zhongshan Road, Dalian, 116023, China.
E-mail: kjyuan@dicp.ac.cn
Dr. P. Jing, Q. Wang
State Key Laboratory of Luminescence and Applications, Changchun Institute of Optics, Fine Mechanics and Physics, Chinese Academy of Sciences, Changchun 130033, People's Republic of China.
Mr. Q. Li
Institute of Atomic and Molecular Physics, Jilin University, Changchun, 130012, People's Republic of China.

To whom the correspondence should be addressed. E-mail: cxshan@zzu.edu.cn, E-mail: kjyuan@dicp.ac.cn

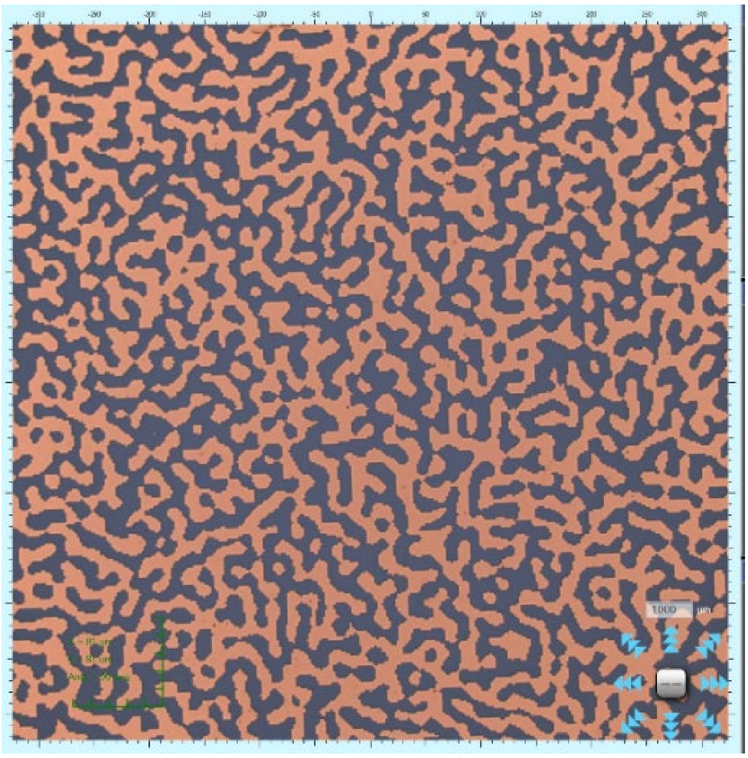

Figure S1. The microstructure of the DOE

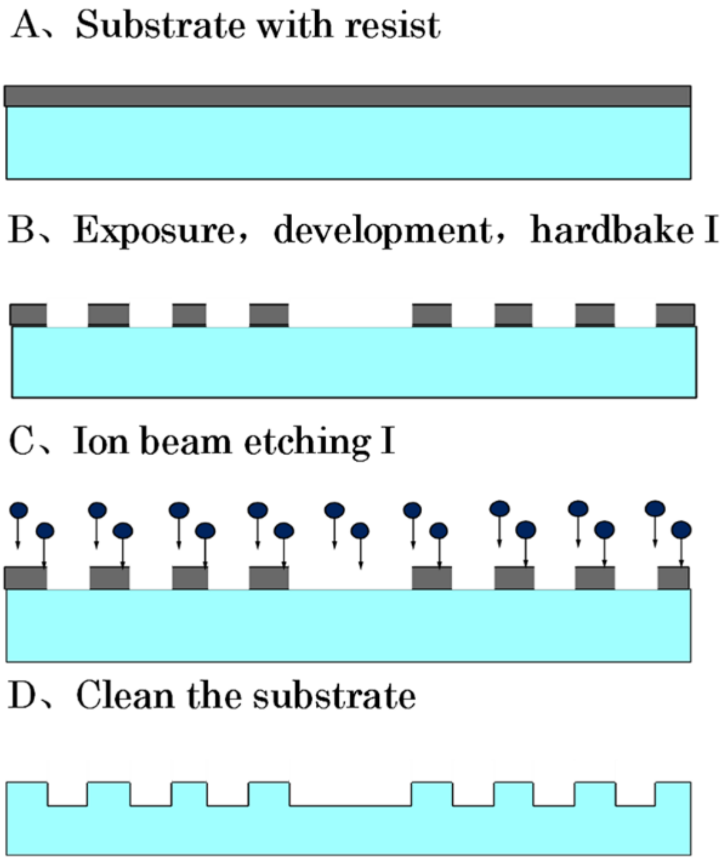

Figure S2. The schematic of DOE’s fabrication process

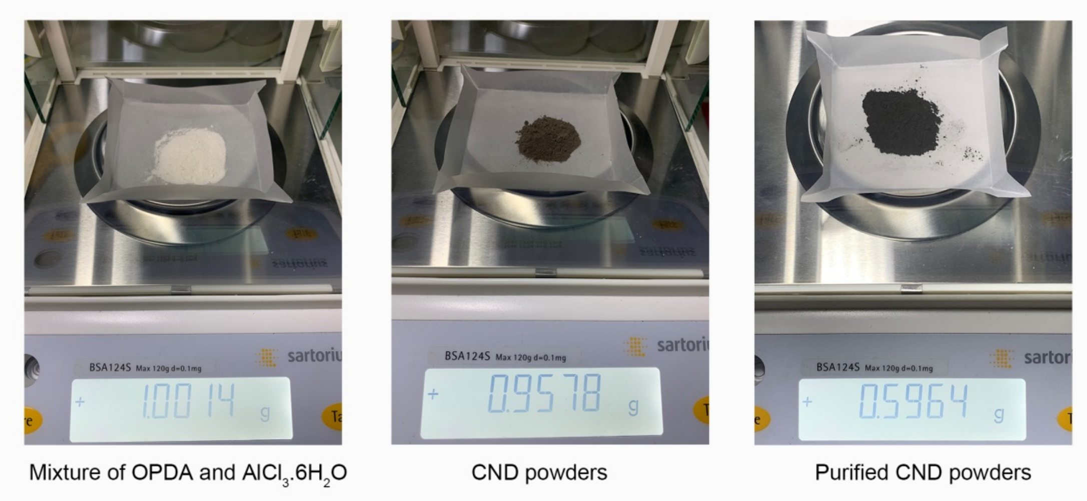

Figure S3. Images of the precursors, CND powders and purified CND powders.

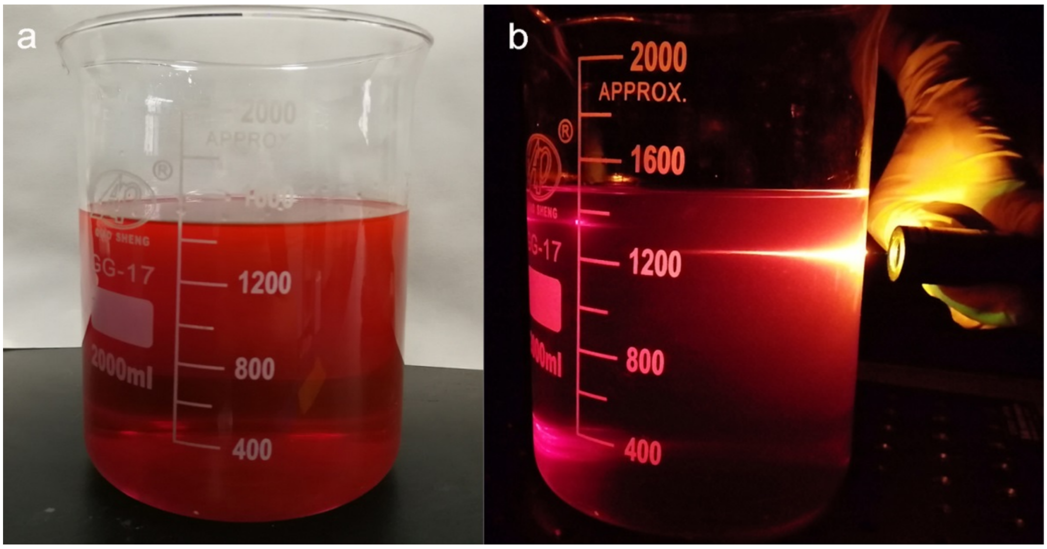

Figure S4. Image of the 1500 ml CND solution under daylight (a) and 532 nm iluumination (b).

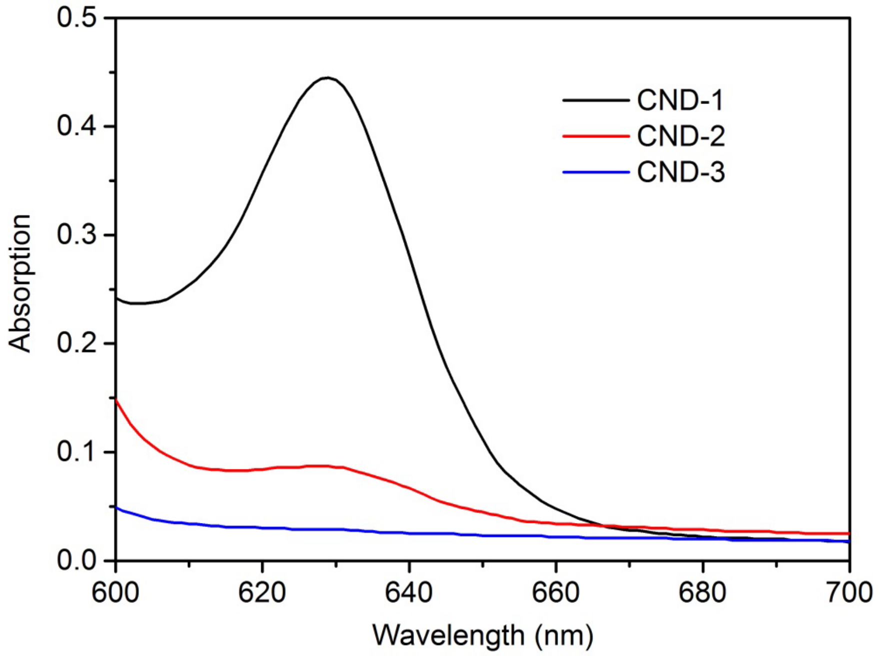

Figure S5. Absorption spectra of CND-1, CND-2 and CND-3

Table R1. The elemental contents of the CNDs

Name	N(%)	C(%)	H(%)	C/N ratio
**CNDs**	24.69	63.57	7.18	2.57

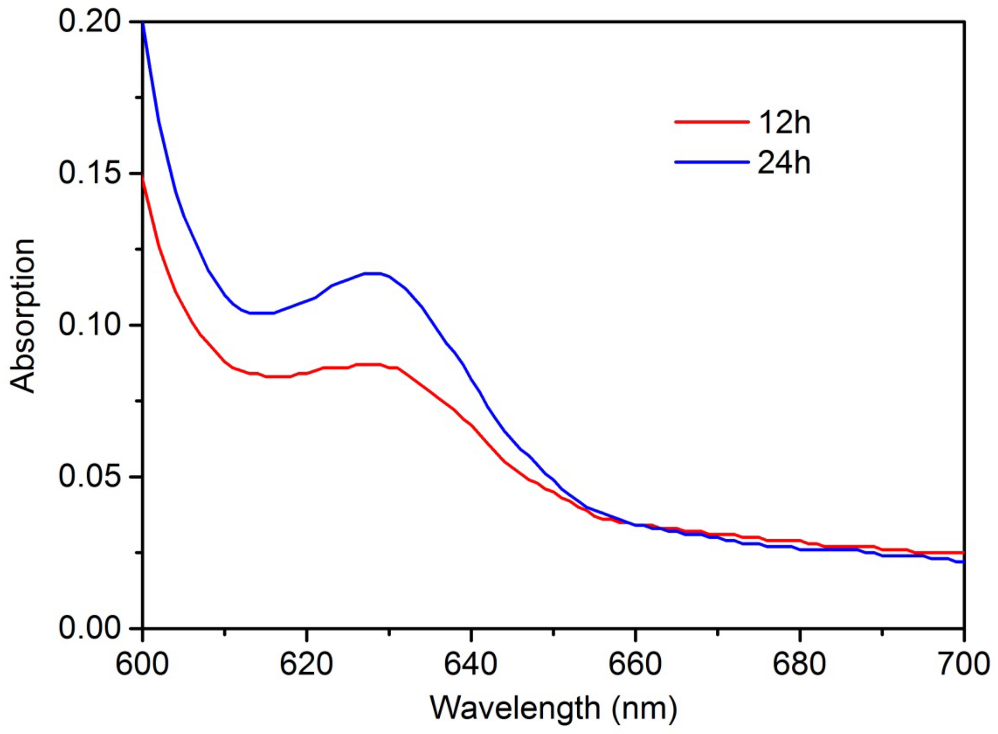

Figure S6. Absorption spectra of CND-2 with treatment for 12 h and 24 h.

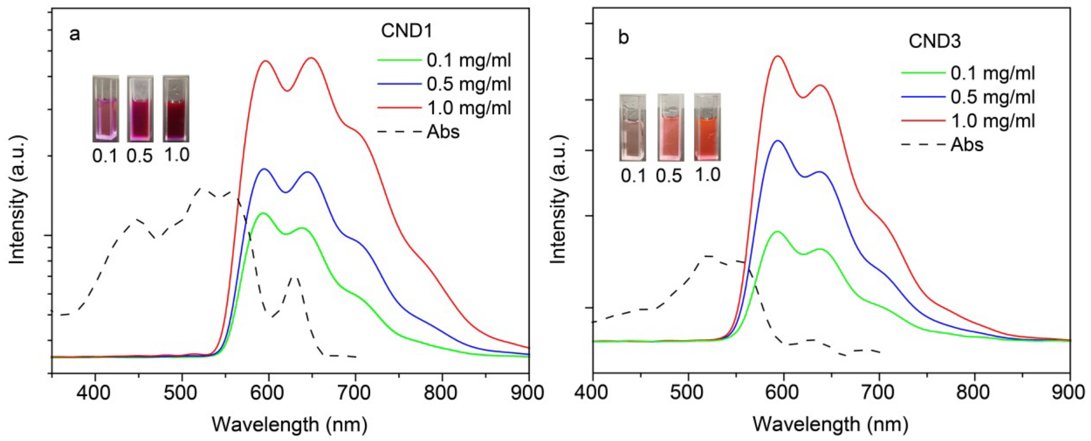

Figure S7. CND-1 (a) and CND-3 (b) PL spectra with concentration from 0.1 to 1.0 mg/ml under 532 nm illumination, the insets are the images with different concentration under daylight.

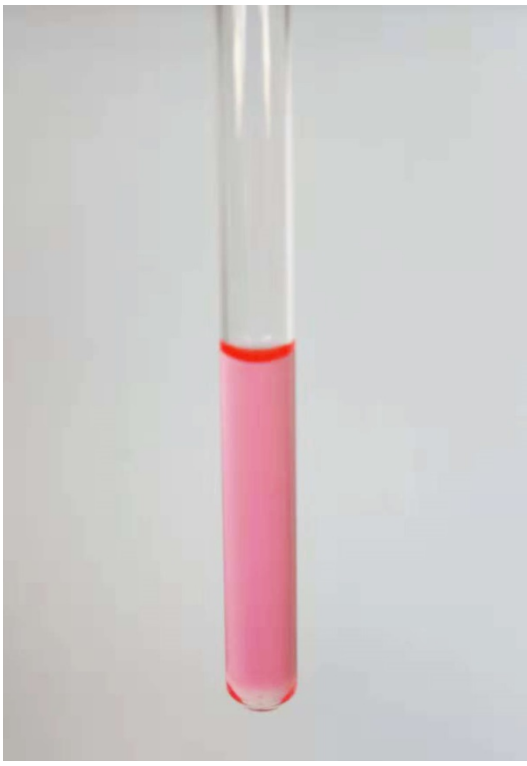

Figure S8. Fluorescence image under daylight excitation.

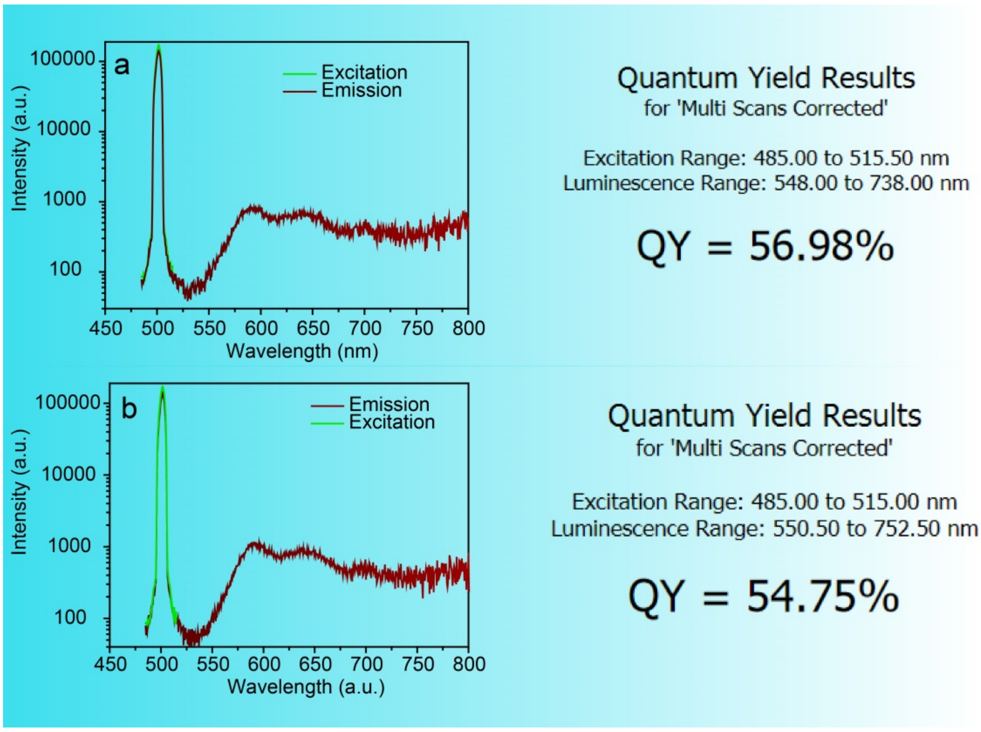

Figure S9. The PL QY of the CNDs

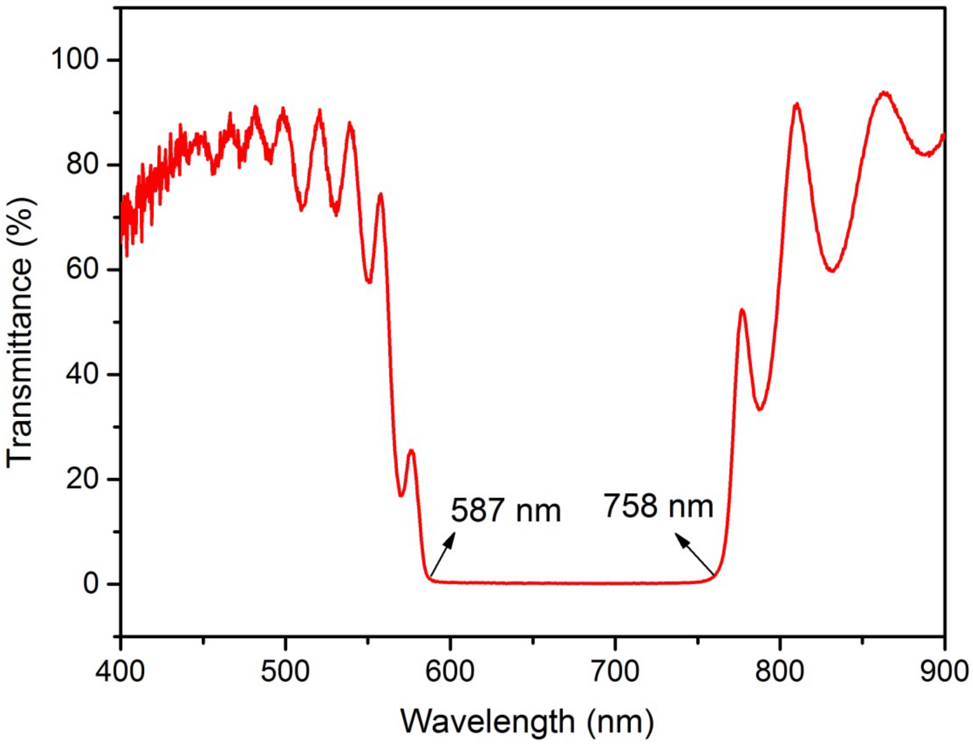

Figure S10. Transmittance spectrum of the band-pass filter

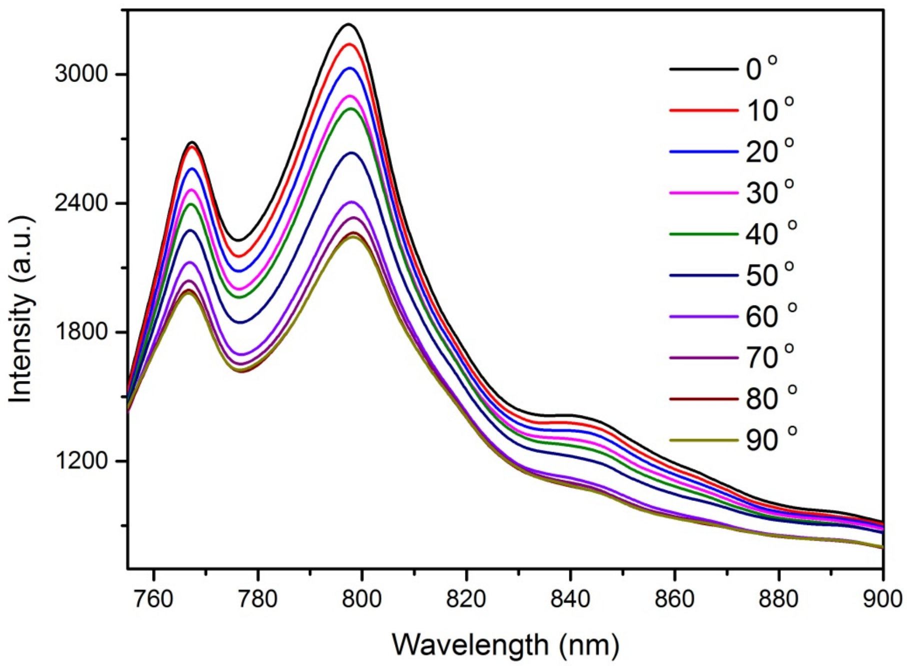

Figure S11. PL spectra of the CNDs at different angels

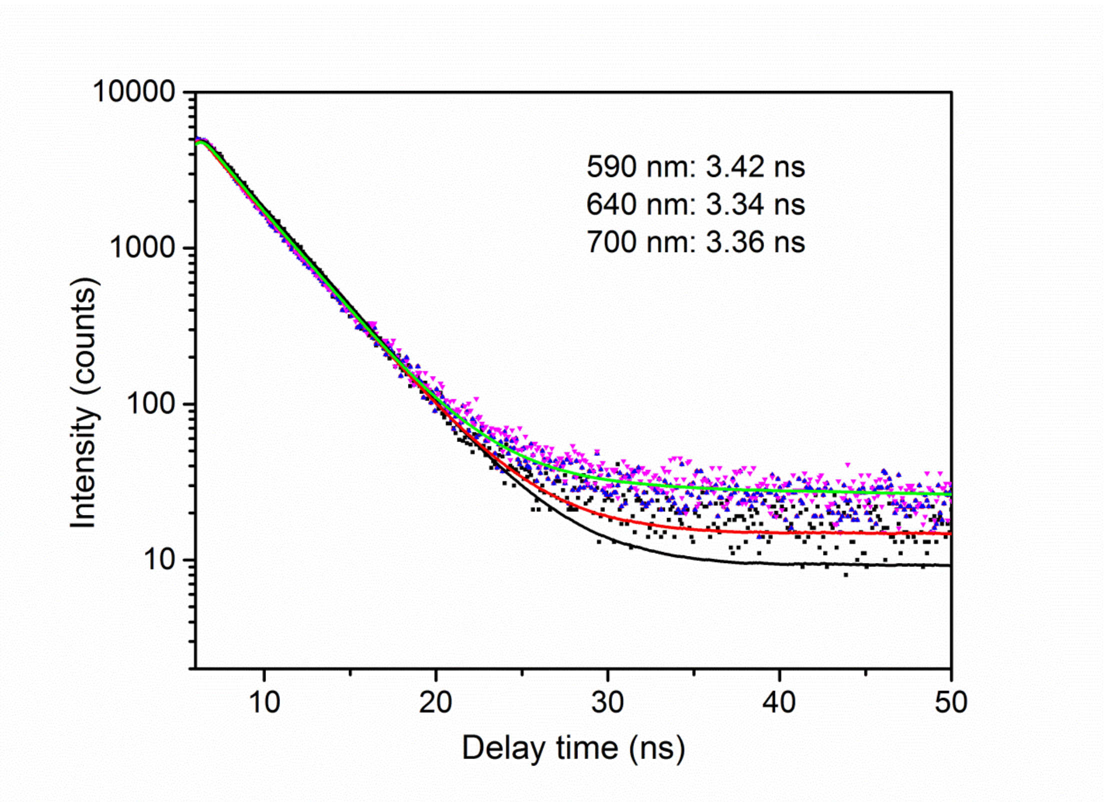

Figure S12. Fitted delay time of the CNDs at different wavelengths

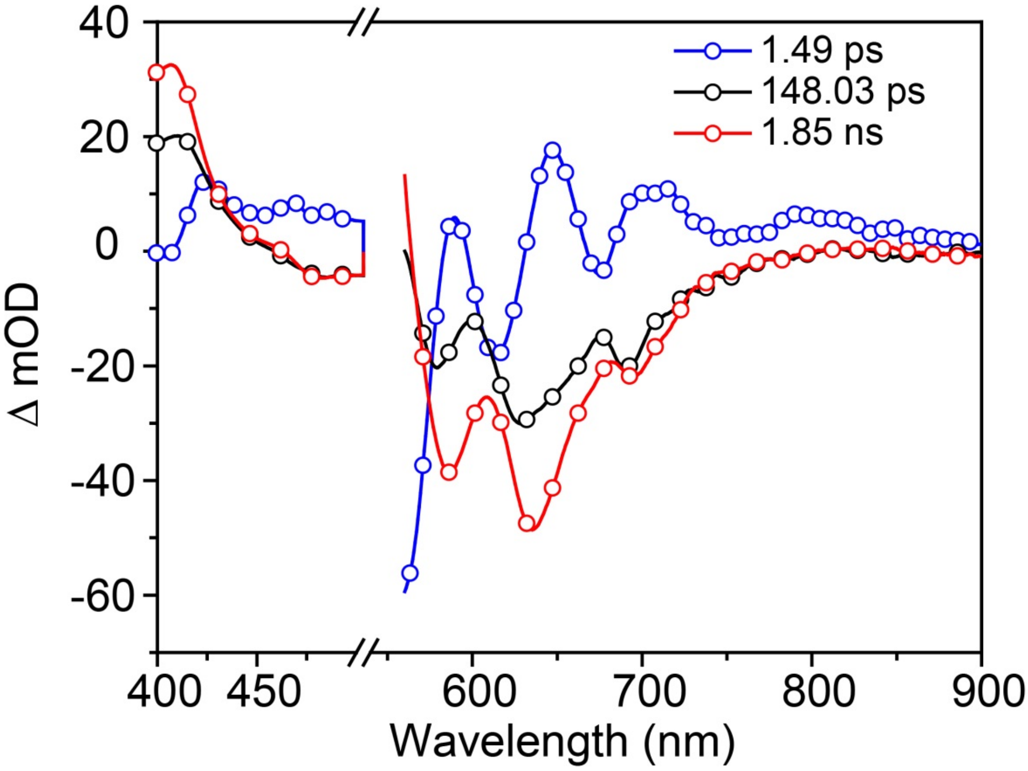

Figure S13. Three species associated SADS of the CNDs

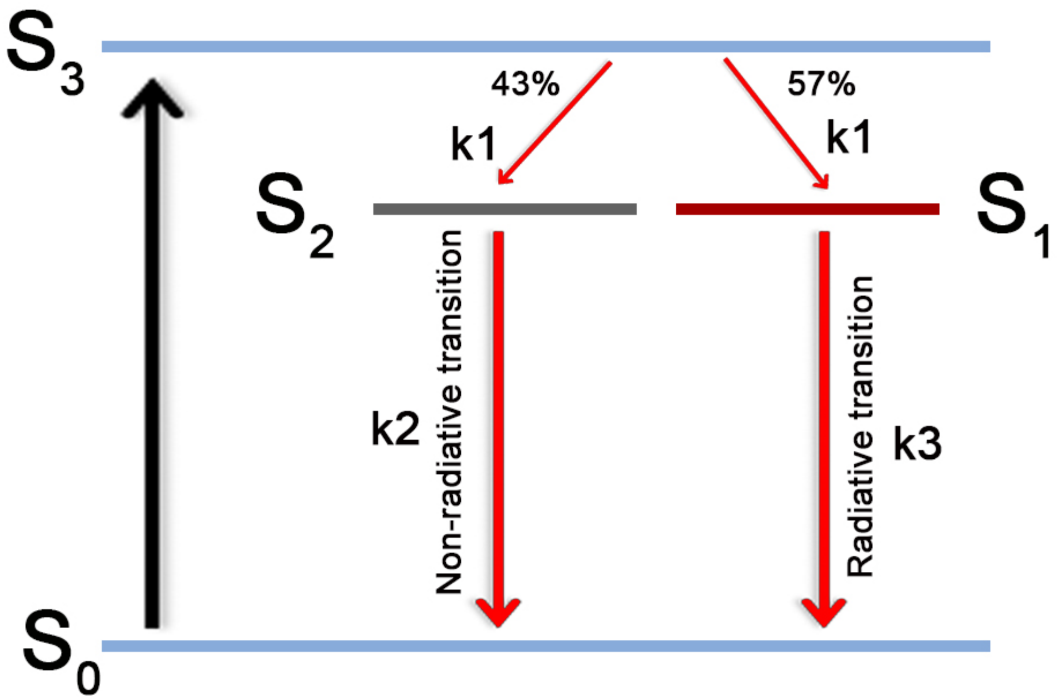

Figure S14. The relaxation and transition model scheme of the excited CNDs. The carriers in different states are characterized by the decay rate (k1, k2, k3). S3: excited state, S2: non-luminous surface state, S1: luminous surface state, S0: ground state. Firstly, the electrons of ground state (S0) will excite into S3 state, then the electrons release to S2 and S1 state via optical photon scattering within 1.49 ps. The PL QY of the CNDs is about 57%, thus 43% electrons are trapped by non-luminous surface state (S2 state), and others are trapped by luminous surface state (S1). The electrons in S2 and S1 state will undergo non-radiative transition (148.03ps) and radiative transition to S0 state (1.85 ns)
